# Physical fitness and physical activity of 6-7-year-old children according to weight status and sports participation

**DOI:** 10.1371/journal.pone.0218901

**Published:** 2019-06-25

**Authors:** Eva-Maria Riso, Lisette Toplaan, Piret Viira, Sille Vaiksaar, Jaak Jürimäe

**Affiliations:** Institute of Sports Sciences and Physiotherapy, Faculty of Medicine, University of Tartu, Tartu, Estonia; University of Brasilia, BRAZIL

## Abstract

**Background:**

The aim of this study was to investigate physical fitness and physical activity (PA) of 6-7-year-old children just before entering school according to their sports club (SC) participation and weight status, and to examine possible associations of their body mass index, fat mass and fat-free mass with physical fitness and PA.

**Methods:**

A total of 256 preschoolers aged 6 to 7 years participated in the study. Physical fitness was assessed using PREFIT test battery and objective PA by 7 day accelerometry. Body composition was estimated using two skinfold thickness parameters. SC participation and parental data were assessed by questionnaires.

**Results:**

Children participating in SC had higher moderate-to-vigorous PA, vigorous PA and cardiorespiratory fitness than children not participating in SC. Overweight children had lower results in cardiorespiratory fitness and all weight-bearing fitness tests, and better results in handgrip strength test in comparison with normal weight children. Significant associations were found between body composition indices and physical fitness tests. PA level was associated with fat-free mass and physical fitness but not with fatness indices.

**Conclusions:**

Weight-status and body composition together with PA level were associated with physical fitness level. The differences in physical fitness based more upon weight status than SC participation among 6-7-years old children.

## Introduction

Physical fitness has been considered a powerful marker of health in children and adolescents [1,2]. However, the reported level of physical activity (PA) and physical fitness of children has decreased during the last decades [3]. Childhood obesity is a serious public health problem globally [4], and is accompanied with accumulation of excess adipose tissue, which may have a negative impact on physical fitness [5]. An inverse cross-sectional association exists between moderate-to-vigorous PA (MVPA) and child adiposity [6]. This is one reason why MVPA is stated as an important factor of paediatric obesity prevention [7] and at least 60 min MVPA per day is recommended to ensure childhood health [4]. Preventive measures such as increased PA performed during 6 to 11 years of life, defined as mid-childhood, could help to anticipate obesity and its adverse effects [8]. Sports club (SC) participation is a modifiable determinant for potential beneficial changes in adiposity, fitness and lifestyle characteristics [3]. Although SC participation starts at the age of 6–7 years in many countries, few data are available about possible differences in physical fitness, body composition and PA level according to SC participation [3]. In the recent study with Swiss children, aerobic fitness differed both according to weight status and SC participation in preschoolers and children not participating in SC were less physically active [3].

Studies conducted in 3-5-year-old children suggest that higher body mass index (BMI) is associated with lower cardiorespiratory fitness [9,10]. As BMI reflects both the fat mass (FM) and fat free mass (FFM) compartments of the body, different associations with physical fitness could be observed [11]. To date, research on the associations between body composition, physical fitness and PA has focused more on older children and/or adolescents [1,12]. Few recent studies have examined associations between body composition, physical fitness and PA in younger preschoolers aged 4-5-years [11,13]. However, we were not able to find any studies that have investigated associations between body composition, physical fitness and PA in 6-7-years-old children. In several countries, 6-7-year-old children already go to school. Thus, it could be proposed that the beginning of school is a big change in their life and might often be connected with changes in daily schedule and PA pattern. Moreover, it is recently stated that PA and physical fitness are low among elementary school children and the proportion of overweight children is high [14]. Thus, it is important to assess possible associations between PA, physical fitness and body composition in just before entering the school.

Accordingly, the aim of this study was to investigate physical fitness and PA of 6-7-year-old children according to their weight status and SC participation, and to examine possible associations of BMI, FM and FFM with physical fitness and PA. In addition, as contradictory data still exist about the interaction of parental education with PA level and physical fitness of children [15], the educational level of parents was used as confounder in analysis of associations.

## Materials and methods

### Participants

The study sample consisted of 13 randomly chosen kindergartens from all 30 municipal kindergartens of Tartu, Estonia. Three hundred ninety parents of the children from the last preschool year groups of selected kindergartens received written information about the study, whereas 284 children with parents agreed to participate. Valid physical fitness indicators, accelerometer data and anthropometrical measurements were obtained from 256 children (132 boys and 124 girls). Parents reported their educational attainment, and this measure was based on the highest level achieved by the mother or father and categorised as university level or less. The parents were also asked whether their child belonged to a SC [16]. Written informed consents were obtained from the parents of all participants. The study was approved by approval 254/T-16, Medical Ethics Committee of the University of Tartu, Tartu, Estonia and was conducted in accordance to Helsinki declaration.

### Physical fitness testing

Physical fitness was tested using four tests within the PREFIT fitness test battery: 20-m shuttle run test for cardiorespiratory fitness, handgrip strength test for upper body muscular strength, standing long jump test for lower body muscular strength, and 4x10-m shuttle run test for motor fitness [17]. The tests used are similar with the tests of ALPHA fitness test battery and this allows to use them in longitudinal studies [18]. All tests were applied twice and the best value of two attempts was used in the analyses, except the 20-m shuttle run test that was conducted only once [17]. The details of the procedures for the physical fitness tests have been described previously [17].

### Measurement of physical activity

The Actigraph GT3X accelerometer (ActiGraph LLC, Pensacola, FL, USA) was used to objectively monitor whole-day PA and sedentary time (SED). Children wore the device on a belt around the waist on the right side for seven days during waking hours, except for during water-based activities [14]. The device was attached to the elastic band. For initiating the accelerometers 30 Hz [14, 19] and normal filters [19] were used [14]. A valid recording for PA and SED required at least 10 hours of recordings for at least three days, including one weekend day [20]. The accelerometer data were analysed using activity counts of 15-second epoch [19; 21]. The night-time periods when the unit was removed and all sequences of 20 minutes or more with consecutive zero counts were removed from analysis [14;20] Time spent sedentary was characterized by <100 counts per minute [22,23]. Activity values between 100 and 1999 counts per minute were registered as light PA. The time spent in moderate PA (MPA) and VPA was calculated based upon the cut-offs of 2000 and 4000 counts per minute, respectively [22,23]. Each individual’s accumulated PA was categorized into different intensities, and average minutes of SED, LPA, MPA and VPA over measured days were subsequently calculated. Time spent in MVPA was calculated as the sum of MPA and VPA. Total PA was calculated as the sum of LPA, MPA and VPA. The children were considered to meet the PA recommendations when the average moderate-to-vigorous PA was 60 minutes or more over all measured days [14,23].

### Anthropometric measurements

Anthropometric measurements were carried out in the kindergarten settings. Body mass and height were measured using calibrated medical digital scales (A&D Instruments, Abington, UK) and portable stadiometer (Seca 213, Hamburg, Germany) to the closest 0.05 kg and 0.1 cm, respectively, with the child wearing light clothing without shoes. Body mass index (BMI) was calculated as body mass (kg) divided by body height squared (m^2^). Age-adjusted BMI cut-off points were used to define overweight and obese subjects [24]. Two skinfold thicknesses (triceps and subscapular) were measured in triplicate on the right side of the body with a Holtain caliper (Crymmych, UK) to the nearest 0.2 mm using standard procedures [25]. In every kindergarten, the same trained investigator made all skinfold thickness measurements. The percentage of body fat (body fat%) and fat mass (FM) were calculated from triceps and subscapular skinfold thicknesses using the Slaughter et al. [26] equations for 6–17 years old children and youth. In addition, fat free mass (FFM) in kg was derived by subtracting FM from total body mass.

### Statistical analysis

Data analysis was made using the SPSS version 20.0 for Windows (SPSS, Inc., Chicago, IL, USA). Descriptive statistics were presented as mean and standard deviations. All variables were checked for normality before the analysis using Kolmogorov-Smirnoff test. All variables presented normal distribution. Group differences between means were analysed with independent t-test, and chi-square test was used to analyse group differences with categorical values. Multiple linear regression models were used to examine the independent associations of body composition (BMI, FM, FFM) values with physical fitness indices (20 m shuttle run, 4x10 m shuttle run, standing long jump, handgrip strength) and PA subcomponents (SED, MVPA, VPA). Standardised β coefficients were used. The variance inflation factors between variables were less than five, suggesting that multicollinearity was not a problem in the models [27]. Three regression models were used: unadjusted model; model adjusted for age and gender of the child (Model 1); and Model 1 adjusted for parental education and participation in SC (Model 2). In addition, the associations between physical fitness indices with SED, MVPA and VPA were further examined. Three regression models were also used: unadjusted model; model adjusted for age and gender (Model 1) and Model 1 adjusted for parental education and participation in SC (Model 2).

Significance was set at p<0.05.

## Results

### Descriptive data of participants

Data of participants according to weight status are demonstrated in Table 1. Significantly more normalweight (NW) children (n = 110; 51%) met daily MVPA recommendations in comparison with overweight (OW) children (n = 14; 34%). More than half of children participated in SC (Table 2). Children not participating in SC had lower (p<0.05) MVPA, VPA, total PA and 20 m shuttle run values compared to children participating in SC. Significantly more children who participated in SC (n = 83; 48%) met daily MVPA recommendations in comparison with children who did not participate in SC (n = 14; 28%) (Table 2). Most popular sports events practised in our study were gymnastics, swimming and soccer.

**Table 1:** Descriptive data of study participants.

	Normal-weight (n = 215)	Over-weight (n = 41)	Whole sample (n = 256)
**Age (yrs)**	6.6 ± 0.5	6.6 ±0.5	6.63 ±0.5
**Height (m)**	1.24 ± 0.05	1.29 ± 0.04*	1.25± 0.05
**Weight (kg)**	23.9 ± 3	31.9 ± 3.4*	25.2± 4.2
**BMI (kg/m^2^)**	15.4 ± 1.0	19.3 ± 1.5*	16.0 ± 1.8
**Fat percent**	19.7 ± 3.5	26.6 ± 4.8*	20.8 ± 4.5
**Fat mass (kg)**	4.7 ± 1.1	8.6 ± 2.7*	5.4 ± 2.0
**FFM (kg)**	19.2 ± 2.4	23.3 ± 2.1*	19.9 ± 2.8
**SED per day (min)**	409 ± 87	416 ± 114	409 ± 91
**LPA per day (min)**	305 ± 42	303 ± 43	305 ± 43
**MPA per day (min)**	48 ± 13	48 ± 17	48 ± 13
**VPA per day (min)**	21 ± 10	18.4 ± 12	21 ± 10
**MVPA per day (min)**	68 ± 22	64 ± 30	68 ± 23
**Total PA per day**	412 ± 158	379 ± 193	411 ± 164
**20 m shuttle run (laps)**	19.5 ± 10	14.7 ± 6*	18.9 ± 9.7
**4x10 m shuttle run (s)**	15.19 ± 1.5	16.04 ± 4.2*	15.32 ± 2.16
**Standing long jump (cm)**	123.5 ± 16.7	113 ± 18.1*	122.0 ± 17.3
**Handgrip strength (kg)**	10.5 ± 2.1	12.2 ± 2.1*	10.9 ± 2.1
**Higher parental education (n; %)**	147; 68	27;66	174; 68
**Meeting MVPA recommendations (n;%)**	110;51	14;34*	124; 48.4
**Participating in organized sport (n; %)**	144; 67	28;68	174; 68

*p<0.05 as compared with normal-weight children

BMI—body mass index; FFM—fat free mass; SED- sedentary time; LPA—light physical activity; MPA—moderate physical activity; VPA—vigorous physical activity; MVPA—moderate-to-vigorous physical activity

**Table 2:** Differences in body composition, physical activity and fitness according to children`s participation in sports club (SC).

Variable	Children with SC participation (n = 174)	Children without SC participation (n = 82)
**Age (yrs)**	6.6 ± 0.5	6.6 ± 0.5
**Height (m)**	1.25 ± 0.05	1.24 ± 0.05
**Body mass (kg)**	25.3 ± 4.3	25.0 ± 4.1
**BMI (kg/m^2^)**	16.1 ± 1.8	16.0 ± 1.7
**Body fat%**	20.5 ± 4.6	21.6 ± 4.3
**FM (kg)**	5.3 ± 2.1	5.5 ± 1.7
**FFM (kg)**	20.0 ± 2.7	19.6 ± 2.9
**SED per day (min)**	408 ± 90	413 ± 95
**LPA per day (min)**	307 ± 42	298 ± 45
**MPA per day (min)**	49 ± 14	45 ± 11*
**VPA per day (min)**	22 ± 11	18 ± 8.3*
**MVPA per day (min)**	70 ± 24	61 ± 21*
**Total PA per day**	433 ± 168	351 ± 140*
**20 m shuttle run (laps)**	20.3 ± 10	15.6 ± 8.2*
**4x10 m shuttle run (s)**	15.25 ± 2.37	15.50 ± 1.56
**Standing long jump (cm)**	122.5 ± 17.5	120.7 ± 17
**Hand grip strength (kg)**	11.0 ± 2.2	10.6 ± 2.0
**Higher parental education (n; %)**	135; 78	39; 48*
**Meeting MVPA recommendations (n; %)**	83; 48	23; 28*

*Significantly different from children with sports club participation; p<0.05.

FM—fat mass; FFM—fat free mass; BMI—body mass index; FFM—fat free mass; SED- sedentary time; LPA—light physical activity; MPA—moderate physical activity; VPA—vigorous physical activity; MVPA—moderate-to-vigorous physical activity

### Associations of physical fitness and physical activity with body composition indices

Higher BMI was positively associated with higher handgrip strength and negatively with the result of 20 m shuttle run in all models studied (Table 3). Both in adjusted and unadjusted analysis, each unit (kg/m^2^) increase in BMI was associated with a 0.34 kg increase in handgrip strength and a decrease in the result of 20 m shuttle run by 0.2 laps. Negative association was also found between BMI and standing long jump in unadjusted model and after adjustment with confounders in SC (Table 3). Increase in BMI by one unit was associated with the worsening result in standing long jump by 0.18 cm. FM was negatively associated with the 20 m shuttle run and standing long jump results in unadjusted model and after adjustment with age and gender (Table 3). Similarly to BMI, each unit of FM was associated with the 0.2 laps lower result in 20 m shuttle run. Further adjustment with parental education and participation in SC attenuated the negative associations of FM with 20 m shuttle run and standing long jump values. Fat free mass was associated with all measured PA and physical fitness indices (Table 3). Each unit of FFM was associated with 0.6 kg higher result of handgrip strength. Positive association was observed between time spent in MVPA and VPA with FFM and negative association time spent in SED with FFM (Table 3).

**Table 3:** Associations of physical fitness and physical activity with body composition values.

	BMI			FFM			FM		
	r^2^	β	p	r^2^	β	p	r^2^	β	p
**20 m shuttle run**	0.042	-0.204	0.003	0.006	-0.077	0.274	0.048	-0.220	0.001
**Model 1**	0.048	-0.219	0.015	0.092	-0.119	0.000	0.049	-0.216	0.017
**Model 2**	0.066	-0.197	0.025	0.113	-0.113	0.001	0.057	-0.187	0.064
**4x10 m shuttle run**	0.013	0.115	0.087	0.001	-0.033	0.630	0.017	0.132	0.055
**Model 1**	0.016	0.124	0.324	0.070	0.017	0.002	0.020	0.124	0.246
**Model 2**	0.042	0.118	0.146	0.092	0.015	0.004	0.035	0.104	0.265
**Standing long jump**	0.013	-0.176	0.009	0.017	0.129	0.062	0.044	-0.210	0.002
**Model 1**	0.034	-0.189	0.055	0.071	0.081	0.002	0.045	-0.203	0.023
**Model 2**	0.057	-0.189	0.047	0.092	0.090	0.004	0.058	-0.191	0.055
**Hand grip strength**	0.117	0.342	0.000	0.380	0.616	0.000	0.091	0.302	0.000
**Model 1**	0.119	0.349	0.000	0.398	0.589	0.000	0.111	0.332	0.000
**Model2**	0.134	0.339	0.000	0.425	0.604	0.000	0.124	0.327	0.000
**MVPA**	0.000	0.016	0.825	0.050	0.224	0.002	0.000	-0.011	0.881
**Model 1**	0.005	-0.001	0.782	0.091	0.193	0.000	0.005	-0.005	0.804
**Model 2**	0.014	-0.004	0.759	0.113	0.217	0.001	0.015	-0.012	0.755
**VPA**	0.000	0.012	0.870	0.042	0.205	0.005	0.002	-0.046	0.531
**Model 1**	0.007	-0.003	0.712	0.096	0.167	0.000	0.007	-0.042	0.735
**Model 2**	0.017	-0.018	0.685	0.097	0.165	0.003	0.020	-0.057	0.615
**SED**	0.000	-0.018	0.797	0.004	-0.060	0.412	0.005	-0.072	0.325
**Model 1**	0.007	-0.008	0.709	0.074	-0.063	0.003	0.009	-0.065	0.630
**Model 2**	0.017	-0.018	0.684	0.075	-0.059	0.018	0.023	-0.075	0.551

Model 1- adjusted with age and gender; model 2 –adjusted with age, gender, parental education and participation in organized sport

FM—fat mass; FFM—fat free mass; BMI—body mass index; FFM—fat free mass; SED- sedentary time; VPA—vigorous physical activity; MVPA—moderate-to-vigorous physical activity

### Associations of time spent sedentary and in different PA levels with physical fitness indices

Associations between time spent in MVPA with 20 m shuttle run and standing long jump were positive in all models studied (Table 4). MVPA was positively associated with 4x10 m shuttle run after adjusting with age and gender (Table 4). The association was no longer significant after adjusting with parental education and participation in SC (Table 4). Handgrip strength was positively associated with MVPA in adjusted models. Positive association was found between VPA and 20 m shuttle run as well as with standing long jump in all models used (Table 4). Each measured unit of VPA was related with 0.14 s better perfomance in 4x10 m shuttle run and with 0.3 cm better standing long jump result (Table 4). Positive association was observed between VPA and 4x10 m shuttle run when adjusted with age and gender. However, the association was not observed after controlling for parental education and participation in SC. Positive association was found between 20 m shuttle run and time spent in SED, but the association may be indirect, while no differences in time spent SED were found between studied subgroups (see Tables 1 and 2). SED time and 4x10 m shuttle run were significantly associated in adjusted models. Each unit of SED time was related with 0.14–0.16 s lower result of 4x10 m shuttle run. SED time and standing long jump were inversely associated in a model adjusted with age and gender (Table 4).

**Table 4:** Associations of physical fitness with sedentary time and different PA levels.

	20m shuttle run			4x10m shuttle run			Standing long jump			Handgrip strength		
	r^2^	β	p	r^2^	β	p	r^2^	β	p	r^2^	β	p
**SED**	0.024	0.155	0.039	0.018	0.134	0.072	0.003	-0.055	0.465	0.004	-0.067	0.374
**Model 1**	0.064	0.181	0.011	0.058	0.146	0.016	0.046	-0.061	0.045	0.043	-0.058	0.056
**Model 2**	0.133	0.183	0.000	0.068	0.164	0.047	0.055	-0.055	0.111	0.060	-0.040	0.078
**VPA**	0.039	0.199	0.008	0.020	-0.142	0.056	0.109	0.330	0.000	0.004	0.066	0.375
**Model 1**	0.060	0.172	0.015	0.054	-0.132	0.023	0.142	0.332	0.000	0.041	0.036	0.066
**Model 2**	0.121	0.149	0.001	0.061	-0.141	0.073	0.162	0.341	0.000	0.030	0.036	0.080
**MVPA**	0.056	0.236	0.001	0.017	-0.130	0.077	0.089	0.298	0.000	0.011	0.106	0.154
**Model 1**	0.074	0.217	0.004	0.054	-0.129	0.022	0.128	0.300	0.000	0.045	0.086	0.045
**Model 2**	0.130	0.177	0.000	0.061	-0.143	0.072	0.144	0.317	0.000	0.067	0.094	0.047

Model 1 –adjusted with age and gender; model 2 –adjusted with age, gender, parental education and participation in organized sport

PA—physical activity; FM—fat mass; FFM—fat free mass; BMI—body mass index; FFM—fat free mass; SED- sedentary time; VPA—vigorous physical activity; MVPA—moderate-to-vigorous physical activity

## Discussion

The main finding of the study was that differences in physical fitness tests were more weight-status related than SC participation related despite of the higher PA level and cardiorespiratory fitness of children participating in SCs. Time spent in MVPA and particularly in VPA was associated with better results of all weight-bearing physical fitness tests, while longer SED time was related with the lower performance in 4x10 m shuttle run and standing long jump tests. Higher FFM was associated with more time spent in MVPA and VPA and better perfomance in standing long jump and handgrip strength, which are the indices of lower and upper body muscular strength, but not with 20 m shuttle run and 4x10 m shuttle run, which reflect cardiorespiratory and motor fitness. Higher BMI and FM were associated with the lower results of weight-bearing physical fitness tests, while no associations were found between BMI and FM with MVPA, VPA and also SED time. In addition, physical fitness has also a relationship with body composition [28]. Differences in physical fitness based both on weight status and SC participation of children were observed in this study. Overweight children and children not participating in SC had significantly lower cardiorespiratory fitness as measured by 20 m shuttle run compared with their NW peers and children participating in SCs. This finding is in agreement with previous results obtained both in 5-year old preschoolers [3] and primary school children [29,30,31]. At the same time, children participating in SCs had higher MVPA level and accordingly they had better endurance to perform such strenuous exercise as 20 m shuttle run test. It is also possible that these children were more familiar to make an effort due to regular participation in extracurricular sports lessons. The worser perfomance of OW children in weight bearing physical fitness tests has also been suggested in several other recent studies [9,10,18]. A higher BMI and FM have also consistently been associated with a lower cardiorespiratory fitness in children [5,32]. The authors propose that as fitness tests were mostly weight bearing, requiring speed of movements and agility, overweight children often dislike such activities and thus their normalweight peers exceed them in running and jumping despite of the similar PA level [9,10]. The study of Artero et al 2010 with adolescents also suggests that overweight and obese adolescents achieve lower performances compared with their normal weight counterparts in all tests requiring propulsion or lifting of the body mass. The excess body fat observed in overweight and obese adolescents could explain these differences, because it is an extra load to be moved while performing the tests [33].

Overweight children had expectedly better results in handgrip strength, which is also reported in previous studies in 3-5-year-old children [11,18]. Weight-related differences in physical fitness of 6-7-year-old children were more expressed in our study than SC participation related differences. In addition, SC participants exceeded the children not participating in SC only in 20 m shuttle run test as NW children were better than OW children in all weight-bearing physical fitness tests.

SC participation represents a modifiable determinant for beneficial changes in physical fitness and lifestyle characteristics already in children at very young age [3]. In the current study, more than half of the study participants were engaged in SCs (68%), and no gender differences in SC participation were registered. However, less than half (48.4%) of all studied children met the current recommendations of 60 min MVPA per day. Although the rate of SC participation was rather high, only 48% of SC members met current MVPA recommendations, which is still significantly more than among children not participating in SCs (28%). The training sessions of a study participants took mostly place twice a week, and in most instances, the participation in SC cannot always ensure sufficient MVPA according to current recommendations of 60 min MVPA per day. The recent study with 11-12-years old Portuguese schoolchildren demonstrated also that the frequency of SC practice is decisive to meet PA recommendations [34]. Organized SC practice at least three times or more weekly has been required to influence the compliance of the recommendation of at least 60 min of MVPA [34]. However, the average daily MVPA level of our kindergarten children participating in SCs was higher compared to children not participating in SC. In agreement with findings in younger kindergarten [3] and older primary school [35,36] children, members of the SCs spent more time in VPA than children not participating in SC in our study. Despite the higher average PA level of SC members we found no SC participation-related differences in body composition indices. The reason for this finding could be explained by a short actual time spent in organized SC participation [30]. In accordance, the report of Ara et al [29] showed that differences in body FM were observed only if the duration of SC activities was at least 3 h per week in prepubertal boys.

Sixteen percent of study participants were considered to be overweight or obese. In contrast to previous studies [37,38], no weight status or SC participation related differences were found in SED time. Similarly to our findings, Collings et al [15] have also not found differences in SED time according to weight status in their study with 4-year-old children. Additionally, in the recent study with 7-9-years old schoolchildren, no difference in PA level and SED time was found based on the weight status of participants [14]. In this study the number of obese primary school children was marginal and this could be the reason of absence of differences in PA level and SED time [14]. The SC participation rate and PA level did not differ between NW and OW children in our study. This result is in accordance with previous studies performed with younger preschoolers [3]. Another prior report in preschoolers found that OW girls participated more frequently in SC, which could be explained by a parental concern about their child’s weight status [37]. Parental knowledge and support towards PA and healthy lifestyle are very important in the life of 6-7-year-old children [39]. The results of our study showed that significantly more SC participants had parents with university degree (78%) as compared with children not participating in SCs (48%). Accordingly, SC participation of preschoolers could demonstrate the parental attitude and family lifestyle [39].

The results of this study also demonstrated that higher BMI and FM were associated with worse perfomance of preschoolers in weight bearing fitness tests such as 20 m shuttle run, 4x10 m shuttle run and standing long jump, although the comparison of time spent in different PA intensity levels between NW and OW children showed no differences. Accordingy, BMI was not associated with time spent in different PA intensities in our study sample. Similar finding was reported in recent study with primary school children [14]. FFM was significantly associated with time spent in MVPA, and VPA being simultanously inversely associated with SED time in children studied. An interesting finding was that the association of FFM and running tests showed unexpected direction as higher FFM value did not enhance the result of tests evaluating cardiorespiratory and motor fitness. Since OW children had significantly higher FFM than NW counterparts, high BMI and FFM together were associated with lower results of 20 m shuttle run and 4x10 m shuttle run. The data about the associations of accelerometer-derived PA with all types of physical fitness in 6-7-year-old preschool children are scarce [13]. Our results revealed that time spent in MVPA and VPA was associated with better results of weight-bearing physical fitness tests and are in accordance with previously published studies concerning associations between PA and fitness tests in younger preschool children [13]. VPA was not associated with upper body strength on the plausible reason that vigorous activities (running, jumping) of children strengthen more muscles of lower body instead of upper body muscles [13]. Handgrip strength as the indicator of upper body muscular fitness was associated only with MVPA in models adjusted with age, gender, parental education and participation of SC. It is not surprising that more SED time was related with lower results in fitness tests being simultanously negatively associated with FFM.

This study has some limitations. Due to the cross-sectional design of the study, causality and direction of associations cannot be concluded. In addition, body composition was measured indirectly using skinfolds. The strength of our study is the use of accelerometers to objectively measure PA, the assessment of physical fitness using the PREFIT test battery [17], and the use of several confounders when analysing independent associations between PA and body composition variables with physical fitness indices. In addition, a specific homogeneous group of Caucasian preschoolers just before entering to school was studied.

## Conclusions

Differences in physical fitness tests were more weight-status related than SC participation related, despite of the higher PA level of children participating in SCs. Higher BMI and FM were associated with lower results of weight-bearing physical fitness tests, but no associations were found between BMI and FM with MVPA and SED time. FFM was positively associated with MVPA and VPA and inversely with SED time. Higher FFM was associated with better results of standing long jump and handgrip strength. Time spent in MVPA and particularly VPA was associated with better results of all weight-bearing physical fitness tests, while longer SED time associated with worse performance in 4x10 m shuttle run and standing long jump. It was concluded that weight-status and body composition together with PA level was more associated with the physical fitness than SC participation in 6-7-year-old preschoolers.

Further studies are needed to assess the tracking of PA patterns and health behaviour in children during transition from kindergarten to school.

## Supporting information

S1 Appendix and Legend (PDF)
