# Supplementary material for: Physical fitness and physical activity of 6-7-year-old children according to weight status and sports participation
Source: PLoS One. 2019 Jun 25;14(6):e0218901. doi: 10.1371/journal.pone.0218901 (PMC6592557; doi:10.1371/journal.pone.0218901)
Supplement: S1 Appendix and Legend — (PDF) [file pone.0218901.s001.pdf]

Subject	Age	Gender	SEDday	MPAday	LPAday	VPAday	MVPAday	MVPAnorm
001	7	2	-	-	-	-	-	-
2	6	1	-	-	-	-	-	-
003	7	2	-	-	-	-	-	-
004	7	1	698,9286	37,71429	337,46429	12,53571	50,25	2
5	7	2	-	-	-	-	-	-
006	6	1	561,8214	86,03571	372,32143	44,28571	130,321	1
007	6	1	343,2917	81,33333	350,5	33	114,333	1
008	6	1	-	-	-	-	-	-
009	7	1	419,5357	32,17857	265,71429	30,42857	62,607	1
010	6	2	324,05	55,05	332,15	33,3	88,35	1
011	7	2	504,8	25,65	190,7	7,35	33	2
012	7	2	372,0833	33,95833	291,95833	12,5	46,458	2
013	6	1	388,7143	44	361,39286	11,85714	55,857	2
014	7	1	335,2143	47,60714	368,07143	13,82143	61,429	1
015	7	1	361,6	64,7	351,65	26,6	91,3	1
016	6	1	304,2083	93	334,79167	56,33333	149,333	1
017	6	1	356,65	41,35	247,15	12,4	53,75	2
018	7	1	430,9583	39,66667	214,33333	16,79167	56,458	2
019	6	1	380,1786	49,82143	355,17857	12,46429	62,286	1
020	6	2	340,1667	45,41667	263,20833	17,70833	63,125	1
021	7	1	-	-	-	-	-	-
022	6	2	332,6786	49,57143	309,5	31,92857	81,5	1
023	7	1	490,8571	37,67857	263	17,67857	55,357	2
024	6	2	327	62,7	316,7	27,75	90,45	1
025	7	1	512,75	38,67857	248,78571	20,64286	59,321	2
026	6	2	383,0625	39,25	246,9375	29,875	69,125	1
027	6	1	429,1071	56,28571	350,85714	27,75	84,036	1
028	7	1	318,9643	71,28571	276,46429	59,67857	130,964	1
029	6	2	570,5357	64,17857	404	12,28571	76,464	1
030	6	1	334,8571	32,21429	320,17857	7,714286	39,929	2
031	7	1	393,5714	44,71429	283	13,07143	57,786	2
032	7	2	395,5357	30,32143	278,89286	12,75	43,071	2
033	7	2	575,4286	73,46429	393,42857	31,53571	105	1
034	7	2	386,1786	43,57143	302,60714	14,5	58,071	2
035	7	1	380,9286	44	310,92857	19,57143	63,571	1
036	7	2	385,8929	39,25	285,64286	19,35714	58,607	2
037	6	1	444,9286	53,17857	237,67857	27,17857	80,357	1
038	6	2	391,9167	37,75	280,08333	14,79167	52,542	2
039	6	1	366,45	46,5	354,6	15,2	61,7	1
040	7	2	297,25	56,625	300,5	12,625	69,25	1
041	6	1	420,375	33,16667	286,83333	19,70833	52,875	2
042	6	2	335,7857	38,35714	308,35714	9,607143	47,964	2
043	7	1	399,6071	56,14286	315,35714	17,14286	73,286	1
044	7	1	382,35	69,35	331,95	22,45	91,8	1
045	6	2	521,7857	29,78571	286,25	16,17857	45,964	2
046	7	2	485,5357	44,92857	286,25	27,28571	72,214	1
47	7	1	-	-	-	-	-	-
48	7	2	-	-	-	-	-	-
49	6	2	-	-	-	-	-	-

50	7	2	-	-	-	-	-	-
51	6	1	-	-	-	-	-	-
052	6	2	-	-	-	-	-	-
053	6	1	-	-	-	-	-	-
054	-	-	314,8929	52,21429	267,46429	28,60714	80,821	1
55	7	1	-	-	-	-	-	-
056	6	2	383,55	58,5	323,65	16,7	75,2	1
057	6	2	381,9643	51,71429	263,35714	28,14286	79,857	1
058	7	1	383,6071	48,25	331	14,46429	62,714	1
059	7	2	413,6429	44,28571	345,39286	17,67857	61,964	1
060	6	2	399,4167	34,45833	285,33333	8,125	42,583	2
061	6	1	390,625	62,04167	323,25	24,41667	86,458	1
062	7	1	431,375	33,45833	288,16667	8,708333	42,167	2
063	7	1	422	47,25	321,41667	12,70833	59,958	2
64	-	-	-	-	-	-	-	-
065	6	2	-	-	-	-	-	-
066	7	1	344,75	42,83333	284,08333	18	60,833	1
067	7	1	371,15	49,2	279,25	18,5	67,7	1
068	7	1	390	47,17857	323,89286	15,92857	63,107	1
069	7	2	393,6071	34,10714	288,10714	10,64286	44,75	2
070	7	2	404,5833	31,875	316,125	10,375	42,25	2
71	-	-	-	-	-	-	-	-
072	7	2	-	-	-	-	-	-
073	6	2	-	-	-	-	-	-
074	6	2	446,125	31	258,66667	13,83333	44,833	2
075	7	1	406,8333	47,95833	274,5	21,20833	69,167	1
76	-	-	-	-	-	-	-	-
77	7	2	-	-	-	-	-	-
078	7	2	356,65	56,3	363,55	19,65	75,95	1
079	7	1	385,9	53,075	331,45	29,8	82,875	1
80	7	1	-	-	-	-	-	-
081	7	1	416,75	23,15	261,15	2,7	25,85	2
082	7	1	389,7	73,2	333,7	33,15	106,35	1
083	7	1	318,2	50,45	331,15	7,75	58,2	2
084	6	2	463,1	50,15	340,6	23,25	73,4	1
085	7	1	404,65	38,85	248,05	16,4	55,25	2
086	7	1	362,05	52,7	287,95	18,5	71,2	1
087	7	1	274,0833	70,58333	354,41667	27,58333	98,167	1
88	7	1	-	-	-	-	-	-
089	7	2	384,85	29,3	270,2	7,5	36,8	2
90	7	2	-	-	-	-	-	-
091	6	1	360,5	43,5625	291,5	15,8125	59,375	2
92	7	1	-	-	-	-	-	-
93	6	1	-	-	-	-	-	-
094	6	1	305,1	62,75	338,15	30,7	93,45	1
95	6	1	-	-	-	-	-	-
96	6	2	-	-	-	-	-	-
97	6	2	-	-	-	-	-	-
098	6	1	414,4583	35,625	222,04167	15,20833	50,833	2
099	6	1	395,1875	39,875	286,0625	11,4375	51,313	2

100	7	1	426,25	36,60714	298,71429	15,32143	51,929	2
101	7	1	-	-	-	-	0	2
102	7	2	510,0833	28,75	234,08333	23,29167	52,042	2
103	7	1	631,2143	44,96429	370,60714	14,39286	59,357	2
104	7	1	-	-	-	-	-	-
105	6	1	349	98,58333	383,04167	47,875	146,458	1
106	7	2	485,75	37,33333	280,75	12,41667	49,75	2
107	7	2	504,5833	40	281,54167	13,08333	53,083	2
108	7	1	339,4583	53,04167	328,375	16,5	69,542	1
109	7	1	356,3571	53,35714	261,53571	24,25	77,607	1
110	7	1	415,6	47,4	308,8	15,85	63,25	1
111	6	2	426,5833	39,79167	228,54167	16,91667	56,708	2
112	6	1	335,2083	51,375	366,08333	13,79167	65,167	1
113	7	1	372,875	39	301,83333	12,04167	51,042	2
114	7	2	452,9583	35,66667	212,125	25,83333	61,5	1
115	6	2	-	-	-	-	-	-
116	7	2	373,85	40,05	300	10,2	50,25	2
117	7	1	390,4643	83,67857	309,67857	54,46429	138,143	1
118	7	1	336,5	52,33333	307,75	20,5	72,833	1
119	7	1	412,3125	43,25	259,6875	41,0625	84,313	1
120	7	2	444	28,55	262,7	14,5	43,05	2
121	6	2	337,9583	38	327,75	12,95833	50,958	2
122	-	-	-	-	-	-	-	-
123	6	2	426,25	33,66667	266,70833	11,875	45,542	2
124	7	2	309,65	59,35	298,8	23,25	82,6	1
125	7	2	-	-	-	-	0	2
126	7	2	383,7083	60,16667	286,66667	25,66667	85,833	1
127	-	-	-	-	-	-	-	-
128	6	2	406,45	29,1	257,7	10,75	39,85	2
129	7	1	354,5833	54,20833	375,54167	15,45833	69,667	1
130	7	1	367,0833	74,125	352,41667	22,16667	96,292	1
131	7	1	360,7	45,7	350,5	14,45	60,15	1
132	6	2	403,1071	37,67857	286,03571	9,107143	46,786	2
133	7	1	406,75	28,14286	315,96429	6,785714	34,929	2
134	6	2	341,2857	33,35714	360,57143	11,78571	45,143	2
135	7	1	352,7917	52,58333	343	22,58333	75,167	1
136	8	2	603,3333	41,45833	278,41667	38,83333	80,292	1
137	7	2	-	-	-	-	-	-
138	7	1	380,5833	37,375	301,125	13,04167	50,417	2
139	6	2	356,9286	51,67857	275,71429	25,64286	77,321	1
140	7	2	397,125	48,625	239,125	22,125	70,75	1
141	7	1	402,6786	47,85714	299,57143	14,89286	62,75	1
142	7	2	440,2143	41,71429	283,53571	19,32143	61,036	1
143	7	1	485,1071	53,85714	302,64286	20,82143	74,679	1
144	7	1	389,3929	48,28571	330,21429	12,71429	61	1
145	7	1	397,3409	50,13636	295,47727	19,29546	69,432	1
146	7	2	754,6875	26,5	336,84375	12,90625	39,406	2
147	7	2	713,8438	35,25	341,65625	11,8125	47,063	2
148	7	1	497,875	75,4375	370,125	30,21875	105,656	1
149	7	1	440,25	65,25	347,25	24,35714	89,607	1

150	-	-	-	-	-	-	-	-
151	7	1	439	44,25	287,3125	26,0625	70,313	1
152	7	2	338,5833	56,33333	415,875	15,33333	71,667	1
153	6	1	428,1667	47,41667	273,29167	18,91667	66,333	1
154	7	1	443,9643	46,71429	273,5	24,67857	71,393	1
155	7	1	426,5357	57,57143	288,28571	21,5	79,071	1
156	6	2	462,8929	32,21429	292,07143	7,392857	39,607	2
157	6	2	-	-	-	-	-	-
158	-	-	-	-	-	-	-	-
159	7	2	445,75	52,75	295,125	23,79167	76,542	1
160	7	1	430,95	45,375	352,05	17,35	62,725	1
161	7	1	450,2917	40,16667	252,33333	18,20833	58,375	2
162	6	1	-	-	-	-	-	-
163	6	2	407,1786	36,07143	290,60714	19,5	55,571	2
164	6	1	364,4643	53,78571	282,82143	14,75	68,536	1
165	7	1	354,3	53,55	295,1	29,55	83,1	1
166	7	1	407,75	51,57143	299,85714	22,35714	73,929	1
167	7	1	486,3	29,6	230	18,7	48,3	2
168	7	2	-	-	-	-	-	-
169	6	1	371,5833	58,16667	357,36111	27,19444	85,361	1
170	6	2	393,8333	48,58333	307,625	12,91667	61,5	1
171	7	1	330,5833	75,45833	304,625	27,95833	103,417	1
172	7	2	-	-	-	-	-	-
173	7	2	364,7917	46	328,83333	19,83333	65,833	1
174	7	2	-	-	-	-	-	-
175	7	2	291,1875	51,25	324,625	46,125	97,375	1
176	7	2	381,1786	47,10714	340,96429	12,96429	60,071	1
177	7	2	404,25	55,25	326,375	23,625	78,875	1
178	7	2	447,5417	70,95833	312,375	38,04167	109	1
179	7	2	901,025	20,05	231,175	4,55	24,6	2
180	7	1	717,4643	35,39286	309,21429	13	48,393	2
181	7	1	363,5833	77,41667	355,75	28,125	105,542	1
182	6	1	667,9643	66,14286	342,03571	32,32143	98,464	1
183	7	2	685,3125	29,8125	366,65625	5,375	35,188	2
184	6	2	392,4286	50,96429	324,35714	17,5	68,464	1
185	7	2	445,125	43,66667	270,95833	11,04167	54,708	2
186	6	2	466,0833	27,41667	271,20833	6,916667	34,333	2
187	6	2	400,6429	56	335,14286	20,92857	76,929	1
188	6	1	-	-	-	-	-	-
189	6	1	357,1071	45,71429	311	14,25	59,964	2
190	7	1	463,6667	24,66667	197,5	10,54167	35,208	2
191	7	2	409,625	33,5	252,41667	12,83333	46,333	2
192	7	2	349,2083	60,08333	334,45833	22,45833	82,542	1
193	7	2	364,2917	45	349,91667	10,375	55,375	2
194	7	1	470,8333	52,625	225,5	31,41667	84,042	1
195	6	1	399,125	44	361,875	17,83333	61,833	1
196	7	2	414,9583	49,91667	304,20833	18,79167	68,708	1
197	6	2	350,6667	61,83333	304,25	13,33333	75,167	1
198	6	2	385,5417	58,66667	337,45833	24,125	82,792	1
199	6	2	348,9	52,55	263,8	20,15	72,7	1

200	6	1	411,35	43,3	280,55	14,5	57,8	2
201	7	2	-	-	-	-	-	-
202	6	1	350,25	60,625	306,375	32,625	93,25	1
203	7	1	339,5714	39,03571	275,67857	21,92857	60,964	1
204	6	2	407,15	46,8	281,75	11,9	58,7	2
205	7	1	344,4583	58,75	300	21,95833	80,708	1
206	6	2	443,6429	38,39286	308,35714	13,14286	51,536	2
207	6	1	401,5	39,5625	270,375	19,5625	59,125	2
208	7	1	420,125	47,91667	236,29167	28,125	76,042	1
209	7	1	562	57,78571	310,17857	65,53571	123,321	1
210	7	1	-	-	-	-	-	-
211	7	1	369,0357	35,28571	348,71429	13,35714	48,643	2
212	7	1	390,875	51,625	294,79167	17,875	69,5	1
213	7	1	-	-	-	-	-	-
214	7	2	353,4	54,35	373,15	23,8	78,15	1
215	6	2	293,9583	40,625	326,79167	9,458333	50,083	2
216	6	2	406	40,5	297,20833	9,833333	50,333	2
217	6	1	305,6786	63,60714	340,60714	33,28571	96,893	1
218	7	2	366,0417	35,04167	268,70833	14,41667	49,458	2
219	7	2	393,05	47,95	271,3	20,4	68,35	1
220	7	2	401,3929	46,46429	322,32143	14,14286	60,607	1
221	7	2	-	-	-	-	-	-
222	7	1	-	-	-	-	-	-
223	7	2	382,15	43,5	348,1	27,7	71,2	1
224	6	2	259,35	67,5	358,7	38,35	105,85	1
225	7	2	486,3	33,1	227,95	11,55	44,65	2
226	6	1	-	-	-	-	-	-
227	7	2	410,65	40,55	266,7	23	63,55	1
228	7	1	394,8	50,8	275,15	20	70,8	1
229	6	1	295,75	37,1	317,8	20,35	57,45	2
230	7	2	313,25	45,6875	269,125	29	74,688	1
231	6	1	463,75	40,875	301,125	28,9375	69,813	1
232	-	-	-	-	-	-	-	-
233	7	2	-	-	-	-	-	-
234	7	2	339,3125	42,625	255,4375	14,0625	56,688	2
235	8	1	251,3333	26,16667	462	18,66667	44,833	2
236	7	1	420,875	55,33333	298,58333	26,70833	82,042	1
237	6	1	430,45	56,65	315,7	22	78,65	1
238	7	1	474,5667	52,03333	255,83333	18,73333	70,767	1
239	6	1	-	-	-	-	0	2
240	7	1	375,7143	55,25	327,64286	20,32143	75,571	1
241	6	2	407,625	43,79167	348,33333	15,79167	59,583	2
242	6	2	302,3	57,3	337,2	29,5	86,8	1
243	6	2	356,625	48,25	304,125	24,0625	72,313	1
244	7	1	367,2083	53,75	347,91667	26,375	80,125	1
245	6	2	533,375	41,54167	288,45833	12,16667	53,708	2
246	7	2	400,9	53,15	259,85	42,05	95,2	1
247	7	1	284,5714	84,17857	280,28571	61,92857	146,107	1
248	6	2	450,0417	51,20833	275,875	35,16667	86,375	1
249	7	2	-	-	-	-	-	-

250	7	1	340,5417	44,33333	280,29167	29	73,333	1
251	7	2	344,9167	57,04167	336,125	31,95833	89	1
252	7	1	-	-	-	-	-	-
253	7	2	348,8333	43,33333	334,95833	23,5	66,833	1
254	7	2	353	58,28571	372,85714	29,35714	87,643	1
255	7	2	531,1667	32,08333	411	5,666667	37,75	2
256	7	2	-	-	-	-	-	-
257	7	2	-	-	-	-	-	-
258	7	1	329,2143	58,78571	343,89286	28,57143	87,357	1
259	7	1	458,75	53,16667	323,75	26,75	79,917	1
260	-	-	-	-	-	-	-	-
261	7	1	312,125	67,1875	349,4375	17,375	84,563	1
262	7	2	527,75	29,8	267	12,75	42,55	2
263	7	2	-	-	-	-	-	-
264	6	1	373,1667	49,375	268,20833	30,125	79,5	1
265	7	2	417,2917	49,45833	261,375	25,20833	74,667	1
266	6	1	344,4167	68,375	323,5	27,66667	96,042	1
267	7	1	761,4583	22,04167	287	7,583333	29,625	2
268	6	2	382,75	42,41667	357,5	15,83333	58,25	2
269	7	1	-	-	-	-	-	-
270	6	1	-	-	-	-	-	-
271	6	1	-	-	-	-	-	-
272	6	2	-	-	-	-	-	-
273	6	1	-	-	-	-	-	-
274	7	1	-	-	-	-	-	-
275	6	1	-	-	-	-	-	-
276	-	-	-	-	-	-	-	-
277	6	2	356,2917	57,375	272,41667	26,20833	83,583	1
278	7	2	-	-	-	-	-	-
279	-	-	-	-	-	-	-	-
280	-	-	-	-	-	-	-	-
281	7	2	378,6667	48,29167	284,875	16,79167	65,083	1
282	6	1	462,0625	40,9375	254,5625	23,3125	64,25	1
815	7	2	330,7857	51,92857	378,03571	14,71429	66,643	1
	-	-	-	-	-	-	-	-
	-	-	-	-	-	-	-	-

Handgrip/kg	20mshuttle/laps	4x10m/sec	Standing longjump/cm	Body weight/kg
11,65	12	14,12	160	22
7,75	42	14,45	108	21
12,65	15	14,14	145	22
9,65	39	14,64	136	22
11,1	22	14,5	120	24
11,15	32	15,22	122	26
12,95	43	14,1	147	26
9,2	30	16,06	105	22
7	18	20,93	83	22
10,1	22	15,12	144	26
8,8	12	15,81	118	23
8,25	22	16,53	117	19
16,35	-	14,37	143	35
9,35	17	15,37	110	23
10,85	22	14,75	123	26
7,75	14	15,23	128	22
7,7	27	16,72	88	23
10,9	15	17,87	106	24
9,3	27	15,84	114	23
9	15	16	104	28
9,45	22	14,97	120	23
9,1	19	14,63	136	23
8,25	14	17,5	90	22
8,85	22	16,82	122	21
11,75	20	14,03	102	25
9,95	36	15,19	120	23
9,9	36	14,31	120	26
11,3	22	16,43	142	26
7,75	12	16,37	98	24
7,75	19	15,75	106	20
12,4	26	15,38	115	34
9,6	22	15,06	88	20
13,5	36	14,12	140	29
10,65	20	17,25	100	24
11,65	12	14,68	122	33
8,75	16	16,88	125	26
11,25	26	16,66	132	20
12,35	11	17,85	120	28
13,05	13	15,53	112	28
7,7	22	16,15	116	22
10,85	13	17,66	130	22
8,05	8	19,58	96	34
9,65	35	16,25	122	23
10,85	14	14,9	94	35
11,35	15	17,16	128	24
8,25	23	15,45	116	25
10,55	22	16,38	126	24
14,5	15	14,81	150	25
-	8	16,77	110	20

10,85	23	16,47	126	27
13,7	10	18,4	101	38
11,4	12	17,63	106	22
9,1	37	15,41	126	21
12,75	39	14	146	-
11,05	12	15,65	125	21
15,85	27	16,47	110	37
10,95	39	17,03	100	29
8,95	12	14,56	124	20
16,4	18	13,15	128	30
7,75	17	16,97	108	17
15	-	15,22	145	27
13	37	14,47	138	26
12,25	18	16,5	98	24
-	-	-	-	-
6,75	16	15,35	120	22
11,45	21	15,81	134	23
11,45	20	15,66	132	28
12,15	21	15,58	132	25
7,5	15	-	111	21
8,2	10	19,12	88	41
-	-	-	-	-
-	-	-	-	26
12,85	22	15,43	93	28
10,75	21	13,88	112	24
10,8	-	13,25	144	23
-	-	-	-	-
-	-	-	-	26
11,75	16	14,72	128	-
-	-	-	-	-
-	-	-	-	-
-	-	-	-	24
12,4	10	13,81	-	28
14,1	13	16,41	108	35
7,9	17	15,5	126	22
11,25	25	13,03	143	29
14,15	10	14,84	138	27
-	-	-	-	22
8,8	-	14,59	130	27
10,1	9	14,88	120	28
11,5	21	14,44	128	23
-	14	13,94	138	24
-	-	-	-	-
8,6	14	16,13	116	19
14,15	6	15,07	118	35
13,25	18	13,53	121	22
8,2	17	15,72	104	18
8,55	17	14,56	96	21
10,6	10	15,19	88	21
11,85	-	14,31	126	23

11,35	13	14,87	118	22
12,7	15	13,91	124	26
9,25	29	13,75	130	20
-	-	-	-	32
7,05	7	19,25	110	20
-	-	-	-	36
8,85	29	13,87	118	24
9,4	29	13,63	120	23
11,15	9	16,41	130	28
11,25	12	13,84	146	26
12,05	30	13,97	114	31
8,75	17	17,65	94	21
-	-	-	-	28
8,8	21	14,68	104	32
9,6	29	16,34	130	22
-	-	-	-	24
16,7	11	15,16	108	38
14,75	52	12,65	158	30
9,7	12	16,28	120	27
7,1	29	14,35	120	27
-	-	-	-	28
9,5	12	16,84	110	26
-	-	-	-	-
-	-	-	-	20
12,25	20	16,91	96	-
14,4	15	14,75	128	30
11,2	15	14,6	124	22
-	-	-	-	-
12,35	12	15,75	100	34
12,95	39	13,94	144	21
-	-	-	-	24
12,15	8	14,22	120	32
8,05	7	18,46	90	22
9,45	10	15,22	122	21
10,3	8	20	110	25
12,15	24	13,36	130	25
-	-	-	-	23
9,5	6	14,84	128	24
13,35	8	13,31	126	26
13,25	8	13,85	138	30
-	-	-	-	-
10,4	28	13,7	136	23
13,7	26	14,03	138	24
11,45	54	13,41	152	26
11,9	52	12,25	144	22
11,45	52	12,84	160	21
12	28	12,91	152	22
11,5	31	13,87	148	22
13,6	33	16,06	135	26
-	-	-	-	-

-	-	-	-	-	
	8,65	4	16,65	112	27
	10,5	12	14,96	120	23
	11,25	6	15,31	106	22
	12,85	39	13,53	140	27
	10,9	39	15,5	138 -	
	6,95	8	14,81	106	23
	9,7	12	15,09	118	24
	11,5	10	15,35	123 -	
-	-	-	-		23
	12,75	14	15,16	125	25
	10,6	16	13,75	120	28
	11,5	12	15,56	127	28
-	-	-	-	-	
	11,15	14	15,28	125	26
-	-	-	-		25
	13,2	12	14,97	110	34
	12,5	20	14,75	140 -	
-	-	-	-		26
	11,45	10	13,56	130	25
	8,8	14	15,6	105	19
-	-	-	-	-	
	13,35	17	14,75	150	27
	8,85	17	15,47	122	21
	11,25	13	17,63	116	31
	12,15	13	15,71	140	26
-	-	-	-		19
	12,05	15	16,15	120 -	
	7,55	10	17,03	122	21
	11,45	9	16,66	96	29
	15,1	12	16,34	145	31
	10,7	20	13,75	126	29
	9,7	46	13,91	124	21
	8,95	22	16,22	135	18
	9,3	20	15,84	110	20
	9,85	20	15,13	110	24
	12,7	9	15,34	100	31
	11,15	22	13,56	140	22
	11,35	7	16,41	106	24
	15,35	30	15,31	124	27
	10,15	15	14,16	112	25
	12,55	17	15,75	120	30
	9,95	16	14,53	140	21
	13,3	16	14,81	116	29
	11,85	19	14,34	140	30
	9,45	16	14,41	134	20
	9,8	18	15,47	100	24
	9,8	16	13,5	134	20
	6,85	16	15,07	106	21
	9,45	16	14,62	102	22

-		-	-	-	24
	13,9	19	13,72	128	34
	7,85	24	13,87	132	23
	11,65	38	12,66	142	24
	9,9	23	14,72	132	27
-		-	-	-	-
	11,15	10	14,87	102	27
	7,05	18	13,87	134	21
-		-	-	-	29
-		-	-	-	34
	12,85	31	13,28	132	28
	10,05	14	13,59	138	22
	13,1	13	13,44	128	29
-		-	-	-	26
	15,1	8	14,22	152	26
	9,7	8	14,25	136	25
-		-	-	-	24
	9,95	9	14,87	122	26
	11,75	14	15,4	130	21
	11,2	14	13,47	122	26
	11,95	10	13,5	120	24
	11,9	10	15,63	110	28
	13,55	33	14,19	142	23
	7,65	28	14,25	97	19
	10,55	12	15,41	125	25
	6,55	8	17,41	97	27
	13,15	12	14,06	134	27
	11,15	28	16,44	135	29
	9,1	19	19	112	32
	9,95	16	16,47	102	19
	10,15	10	14,69	130	24
	11,45	27	14,18	158	23
-		-	-	-	-
	10,2	22	14,43	110	22
	10,5	12	14	136	28
	12,35	17	13,94	100	23
	11,35	26	14,87	145	22
-		-	-	-	34
-		-	-	-	21
	8,55	6	14,62	136	23
	10,4	17	17,37	136	22
-		-	-	-	24
	9,7	24	13,16	125	24
	8,7	20	17,44	100	26
	13,7	26	13,37	140	23
	9,9	-	39	57	28
-		-	-	-	23
	11,4	9	14,88	-	30
	11,25	23	15,15	140	24
-		-	-	-	-

	6,3	9	16	82	23
	12,8	14	13,5	142	27
-	-	-	-		22
	14,75	10	13,41	126	26
	8,8	12	16,63	100	22
-	-	-	-		21
-	-	-	-		26
	12,85	20	15,78	126	26
	13	12	15,59	128	28
	10,25	20	15,88	145	25
-	-	-	-	-	
	7,85	18	15,82	122	22
	7,6	10	14,78	100	22
	14,25	11	15,12	112	32
	10,4	28	13	128	20
-	-	-	-		24
	16,1	14	14,87	145	32
	6,35	3	21,94	66	18
-	-	-	-		24
	11,85	22	14,78	126	28
	12,25	28	15,22	136	24
	13,8	11	13,54	135	31
	8,7	8	15,63	125	22
	11,45	11	14,53	150	31
	11,15	16	16,09	115	28
	9,2	22	14,97	124	22
-	-	-	-	-	
	12,3	16	14,38	126	23
-	-	-	-		29
-	-	-	-	-	
-	-	-	-	-	
	11,85	19	13,97	128	21
-	-	-	-		26
-	-	-	-		20
-	-	-	-	-	
-	-	-	-	-	

Body height/cm	KMI	Biceps	Triceps	Subscapulare	Iliac	Fatperc	Fatmass
1,23	14,541609	4	8	4,5	8,2	17,09375	3,760625
1,17	15,340785	6,3	8,7	5	9	18,28103	3,839016
1,23	14,541609	3,5	6,2	4	5	14,71348	3,236966
1,25	14,08	3,5	5,5	5	5	15,03175	3,306985
1,28	14,648438	4	8	4,3	7,3	16,89223	4,054135
1,22	17,468422	6,7	13	8	11,3	24,697	6,42122
1,26	16,376921	3	6	4	5,8	14,5	3,77
1,22	14,780973	4	8	6	10	18,572	4,08584
1,18	15,800057	7	11,7	6	6,8	21,96823	4,833011
1,28	15,869141	6	10,8	7	13,8	22,05508	5,734321
1,23	15,202591	5,2	7	5,2	9	16,79108	3,861948
1,17	13,879757	5	13,3	4,3	4,8	21,88112	4,157413
1,36	18,92301	7,5	13	8,3	16,8	24,93103	8,725861
1,29	13,821285	5,5	10,5	6,7	10	21,53008	4,951918
1,27	16,120032	4	8	4,5	7,2	17,09375	4,444375
1,23	14,541609	8	10	6,3	12,7	20,72503	4,559507
1,22	15,452835	8	10,8	6	8,7	21,17488	4,870222
1,19	16,947956	4,7	9	6,5	11,7	19,99175	4,79802
1,25	14,72	6,8	10,7	7	11	21,96823	5,052693
1,26	17,636684	14,5	21,7	14	23	33,41263	9,355536
1,24	14,958377	5	7,2	4,3	7	16,07575	3,697423
1,22	15,452835	7	10,8	6	12	21,17488	4,870222
1,2	15,277778	6,2	9,5	6	10,7	19,99175	4,398185
1,18	15,081873	5	8,8	5	7,5	18,37828	3,859439
1,28	15,258789	5	7	5	7	16,588	4,147
1,24	14,958377	3,5	8	5,5	10	18,08575	4,159723
1,24	16,909469	5,8	9	5,8	6,5	19,33648	5,027485
1,29	15,624061	3,5	8	5,2	6,5	17,79088	4,625629
1,2	16,666667	7,5	13	6,5	11,2	23,49175	5,63802
1,17	14,610271	7	9	5	6,8	18,572	3,7144
1,36	18,382353	12,2	19	18	23	33,913	11,53042
1,24	13,007284	4	9	4,2	6	17,79088	3,558176
1,33	16,394369	7,3	11,2	7	13,7	22,39988	6,495965
1,28	14,648438	6,8	9	5	9,8	18,572	4,45728
1,34	18,378258	9,2	10,5	7	14,5	21,79375	7,191938
1,29	15,624061	6,5	8	4,5	8,3	17,09375	4,444375
1,19	14,123296	3,7	7	6	8	17,593	3,5186
1,35	15,363512	6,7	11	7	12	22,228	6,22384
1,26	17,636684	6,8	12	11	15,7	26,213	7,33964
1,22	14,780973	8	9	8	11,7	21,353	4,69766
1,26	13,857395	4	6	4	3	14,5	3,19
1,24	22,112383	14,3	18	14,7	24	32,09023	10,91068
1,27	14,260029	3,5	6,7	4	7,5	15,24263	3,505805
1,35	19,20439	12,3	19,2	17,5	23,7	33,80143	11,8305
1,22	16,124698	6	11	6,2	13	21,53008	5,167219
1,22	16,79656	5	11	5,7	8	21,08543	5,271357
1,24	15,608741	6	8	6	6,8	18,572	4,45728
1,23	16,524555	5,3	9,8	6	7	20,26868	5,06717
1,18	14,363689	5	9	6	9,7	19,525	3,905

	1,26	17,006803	9,3	13,3	10	15	26,43143	7,136486
	1,28	23,193359	17	20,3	20	34,8	34,98583	13,29462
	1,17	16,071298	8	15,3	6,5	9,7	25,31588	5,569494
	1,19	14,829461	5,2	8	4	5,7	16,588	3,48348
-	-	-	-	-	-	-	-	
	1,23	13,880627	4,2	7,5	5	8	17,09375	3,589688
	1,34	20,605926	15,3	24,5	20	29,8	35,94175	13,29845
	1,32	16,64371	7,8	13,8	8,7	13,8	25,84375	7,494688
	1,19	14,123296	4,5	7,5	4	6,8	16,07575	3,21515
	1,34	16,707507	5,5	8	7	10	19,525	5,8575
	1,1	14,049587	3,8	5,5	4	6,8	13,96175	2,373498
	1,29	16,224986	5	9	8,8	14,2	22,05508	5,954872
	1,26	16,376921	6	8,8	5,2	10	18,572	4,82872
	1,26	15,117158	5	8	5	5	17,593	4,22232
-	-	-	-	-	-	-	-	
	1,18	15,800057	6	11,3	10,8	8	25,54367	5,619607
	1,23	15,202591	4,7	6,2	5,2	5,5	15,97252	3,67368
	1,33	15,829046	5	7	4,5	6	16,07575	4,50121
	1,28	15,258789	5,2	9	5	7	18,572	4,643
	1,22	14,10911	6,2	11,8	6	8	22,05508	4,631567
	1,32	23,530762	18	27,7	22	30	36,48983	14,96083
-	-	-	-	-	-	-	-	
	1,29	15,624061	8,2	10,2	5,8	10,8	20,452	5,31752
	1,26	17,636684	9,7	11,5	9	19,2	24,30175	6,80449
	1,3	14,201183	7,8	9,2	5,5	7	19,24183	4,618039
	1,22	15,452835	5,2	7,2	5,2	8,8	16,99312	3,908418
-	-	-	-	-	-	-	-	
	1,21	17,75835	9	11	6,8	16	22,05508	5,734321
-	-	-	-	-	-	-	-	
-	-	-	-	-	-	-	-	
-	-	-	-	-	-	-	-	
	1,26	15,117158	7,8	8	5	13	17,593	4,22232
	1,3	16,568047	7,2	9,7	6,8	9	20,90575	5,85361
	1,28	21,362305	13	15	14	31	30,137	10,54795
	1,21	15,026296	8	9,8	6,2	9,2	20,452	4,49944
	1,26	18,266566	7	11	7,2	9,5	22,39988	6,495965
	1,34	15,036757	3,5	6	4,5	9	15,03175	4,058573
	1,21	15,026296	7	7,8	5	8	17,39408	3,826698
	1,32	15,495868	6	9,7	5,2	10,8	19,43087	5,246335
	1,33	15,829046	7,8	11,2	8,5	15	23,65583	6,623632
	1,27	14,260029	5,2	8,2	6	7,5	18,76468	4,315876
	1,29	14,42221	3,5	5,8	4	5	14,28548	3,428515
-	-	-	-	-	-	-	-	
	1,14	14,619883	6	7	4,5	6,5	16,07575	3,054393
	1,3	20,710059	10,3	14,2	13,8	26,7	29,548	10,3418
	1,2	15,277778	3,5	6	4	5	14,5	3,19
	1,17	13,149244	4	7	4	4,8	15,557	2,80026
	1,18	15,081873	7,7	11	6,2	12	21,53008	4,521317
	1,19	14,829461	5	7	6	6,5	17,593	3,69453
	1,2	15,972222	3,8	6,5	5	7	16,07575	3,697423

1,25	14,08	6	8	4,5	5	17,09375	3,760625
1,26	16,376921	4	7	5	7	16,588	4,31288
1,22	13,437248	4,2	5	4	4,5	13,417	2,6834
1,33	18,090339	8	11	7,8	11,5	22,90928	7,33097
1,21	13,660269	3,2	6	4	7	14,5	2,9
1,36	19,463668	8	11,8	7,2	17	23,077	8,30772
1,19	16,947956	10,7	12,5	9,8	12,2	25,69423	6,166615
1,2	15,972222	9	12	8	15	23,9	5,497
1,26	17,636684	8	12,2	8	17	24,06148	6,737214
1,27	16,120032	4,2	8	5,2	11	17,79088	4,625629
1,29	18,628688	10	13	9,8	18	26,06608	8,080485
1,2	14,583333	7	10,8	7,8	11	22,74052	4,775509
1,3	16,568047	4	7	4,8	7	16,38388	4,587486
1,27	19,84004	9,8	12	9,8	15	25,31588	8,101082
1,24	14,308012	6,2	7	5	5,2	16,588	3,64936
1,26	15,117158	4,5	9	5	8,2	18,572	4,45728
1,37	20,246151	8,5	16	9	20,5	27,625	10,4975
1,35	16,460905	5	8,8	5,8	8,8	19,14692	5,744076
1,28	16,479492	8	12,2	8	15	24,06148	6,4966
1,28	16,479492	4,7	8	4,8	7,8	17,39408	4,696402
1,24	18,210198	7	12,2	8	15,2	24,06148	6,737214
1,28	15,869141	9	13,2	9	15,2	25,61908	6,660961
-	-	-	-	-	-	-	-
1,23	13,219644	4,8	6,2	4	5	14,71348	2,942696
-	-	-	-	-	-	-	-
1,27	18,600037	10	14,8	15,7	27,2	30,97175	9,291525
1,21	15,026296	7,8	8,8	7,2	10,2	20,452	4,49944
-	-	-	-	-	-	-	-
1,3	20,118343	14,8	22,8	16,2	26,7	34,597	11,76298
1,19	14,829461	5	6,5	3,5	6	14,5	3,045
1,29	14,42221	5	8	4	6	16,588	3,98112
1,31	18,646932	12	16,2	13	23,5	30,25168	9,680538
1,18	15,800057	10	13	14	16,5	28,933	6,36526
1,16	15,606421	7,2	8,8	5	7,5	18,37828	3,859439
1,23	16,524555	8,2	12	8	19	23,9	5,975
1,24	16,259105	4	7,8	4,8	10,3	17,19412	4,29853
1,27	14,260029	4	8	5	9,8	17,593	4,04639
1,19	16,947956	8,8	12,2	6,8	20,5	23,077	5,53848
1,26	16,376921	7	10,2	7,8	12,8	22,228	5,77928
1,32	17,217631	7,8	11	6,5	16,2	21,79375	6,538125
-	-	-	-	-	-	-	-
1,21	15,709309	6,2	9	6,2	8,5	19,71248	4,53387
1,22	16,124698	4,3	10	6	8	20,452	4,90848
1,27	16,120032	5	6,8	7,8	10,5	19,14692	4,978199
1,22	14,780973	3,2	7	4,2	4,2	15,76528	3,468362
1,22	14,10911	3	4	3	3	11,173	2,34633
1,25	14,08	4	6	3,8	4,8	14,28548	3,142806
1,25	14,08	4	6,5	4	5	15,03175	3,306985
1,34	14,47984	3	5	4	4,2	13,417	3,48842
-	-	-	-	-	-	-	-

-	-	-	-	-	-	-	-
1,27	16,740033	10,2	11,8	10	20,8	25,31588	6,835288
1,26	14,487276	8	10,5	6,2	10,7	21,08543	4,849649
1,23	14,541609	6	7	5	9	16,588	3,64936
1,31	15,733349	7	8	6	9	18,572	5,01444
-	-	-	-	-	-	-	-
1,2	15,972222	5	8	-	-	-	-
1,22	16,124698	8,3	13,2	6,2	13,7	23,40932	5,618237
-	-	-	-	6,5	16,2	-	-
1,26	14,487276	5,5	7,2	-	-	-	-
1,28	15,258789	4,2	7,2	5,5	7,2	17,29423	4,323557
1,23	18,507502	10,8	9,8	5	5,2	19,33648	5,414214
1,28	17,089844	6,8	6,8	9	17,8	20,26868	5,67523
-	-	-	-	5,8	8	-	-
1,27	16,120032	7	8,8	-	-	-	-
1,28	15,258789	5,8	7,5	5	10,2	17,09375	4,273438
1,31	19,812365	10,2	13,8	4,5	11	22,48543	7,645046
-	-	-	-	9	22,2	-	-
1,26	16,376921	9,2	12,8	-	-	-	-
1,26	15,74704	6	7,8	7,2	15	19,525	4,88125
1,14	14,619883	5,2	7	5,2	11,7	16,79108	3,190305
-	-	-	-	5	5,3	-	-
1,29	16,224986	6	8	-	-	-	-
1,24	13,657648	6	8	5,8	8	18,37828	3,859439
1,31	18,064215	9,8	12	4,2	6	20,63428	6,396627
1,24	16,909469	6,8	9	8	18,2	21,353	5,55178
1,15	14,36673	8	9	5,5	7,8	19,05175	3,619832
-	-	-	-	5	7,5	-	-
1,21	14,343283	5	6	-	-	-	-
1,22	19,48401	9,8	16	4,2	7,2	24,06148	6,977829
1,33	17,525016	6	10	10,7	22,7	24,46063	7,582795
1,27	17,980036	6	10	6,2	10	20,63428	5,983941
1,16	15,606421	4,8	7	7	12	18,572	3,90012
1,12	14,34949	4,8	8,8	6	5	19,33648	3,480566
1,16	14,863258	5	6,5	5,5	6,8	16,588	3,3176
1,26	15,117158	7,8	11,8	4	6	20,26868	4,864483
1,25	19,84	10,2	19	8	12,5	28,933	8,96923
1,21	15,026296	4,7	8	20	27	29,548	6,50056
1,23	15,863573	6	10,2	4	5	18,76468	4,503523
1,27	16,740033	5	9	6	6,8	19,525	5,27175
1,19	17,65412	8	15	6	8,8	24,697	6,17425
1,25	19,2	12,8	16	6,8	10	26,06608	7,819824
1,21	14,343283	5,8	9	13	20,7	25,468	5,34828
1,29	17,426837	8	13	6,5	9	23,49175	6,812608
1,28	18,310547	7,5	14,8	10	23	27,48848	8,246544
1,16	14,863258	3	7	6,2	18	17,79088	3,558176
1,22	16,124698	6,8	10,2	4	4,8	18,76468	4,503523
1,22	13,437248	3	6,8	6	11	17,39408	3,478816
1,14	16,158818	5,8	9	4	6	17,593	3,69453
1,2	15,277778	7	10	4,5	9,3	19,05175	4,191385

1,24	15,608741	5	8,5	6	8	19,05175	4,57242
1,27	21,080042	12,2	15	4	6	23,077	7,84618
1,26	14,487276	6	8,7	14,8	22,8	26,57575	6,112423
1,22	16,124698	6,7	11	5	9	20,452	4,90848
1,27	16,740033	10,3	11,5	7	10,8	22,65575	6,117052
-	-	-	-	11	17	-	-
1,3	15,976331	6,5	13	-	-	-	-
1,18	15,081873	4,5	8	8	9,7	20,452	4,29492
1,32	16,64371	6,8	12	4,5	6	20,90575	6,062667
1,38	17,853392	4	8,5	7	18	19,99175	6,797195
1,3	16,568047	6	7,5	6,2	12,7	18,28103	5,118688
1,21	15,026296	3,8	-	6,2	7	-	-
1,32	16,64371	6,8	6,8	6	7,8	17,39408	5,044283
1,33	14,6984	3	14	7	15,3	24,697	6,42122
1,32	14,921947	6,3	5	3	4	12,308	3,20008
1,25	16	9,2	11,2	8,3	16	23,49175	5,872938
1,23	15,863573	7	11	6	14,7	21,353	5,12472
1,23	17,185538	5	10	7	9	21,353	5,55178
1,24	13,657648	4	8	6	11	18,572	3,90012
1,27	16,120032	7	7	5	6	16,588	4,31288
1,3	14,201183	6,8	11,2	6	10	21,53008	5,167219
1,22	18,812147	12	9	6	12,5	19,525	5,467
1,21	15,709309	4	18,8	18	25	33,83888	7,782942
1,14	14,619883	6	7,8	4	8	16,38388	3,112937
1,27	15,500031	7	11	5	6	20,452	5,113
1,27	16,740033	11	15,8	8	13	26,79028	7,233376
1,33	15,263723	6	9	15	18,7	26,932	7,27164
1,33	16,394369	6,3	11	5	9	20,452	5,93108
1,34	17,821341	7,5	10,5	7	16	21,79375	6,974
1,23	12,558662	3,2	5	7	12,7	16,588	3,15172
1,22	16,124698	6,8	11,2	3	4	18,76468	4,503523
1,25	14,72	4,5	8,8	7,7	13	20,90575	4,808323
-	-	-	-	5,8	9	-	-
1,19	15,535626	6,2	10	-	-	-	-
1,31	16,316065	10,8	13	6,8	9	23,73748	6,646494
1,24	14,958377	4,2	6,8	6,8	11	18,18352	4,18221
1,27	13,640027	3	5	4	6,5	13,417	2,95174
1,39	17,597433	9,2	13	4	4	21,353	7,26002
1,21	14,343283	4,2	7	7	18,7	18,572	3,90012
1,2	15,972222	6	8,2	6,8	8	19,525	4,49075
1,23	14,541609	5,5	8,8	7	9	20,26868	4,45911
1,22	16,124698	6	9	7	9	20,452	4,90848
1,25	15,36	6,5	9,2	7	11	20,63428	4,952227
1,23	17,185538	8	13	5,5	7	22,65575	5,890495
1,23	15,202591	3,5	7	8	11,7	19,525	4,49075
1,26	17,636684	8	13	5	5	22,228	6,22384
1,24	14,958377	5,2	9	5	12,2	18,572	4,27156
1,24	19,510926	8,3	12	7,2	7,2	23,24368	6,973104
1,26	15,117158	5,2	8,2	8	20	20,63428	4,952227
-	-	-	-	6	8	-	-

1,22	15,452835	5	10	-	-	-	-
1,25	17,28	7	9	5,2	6	18,76468	5,066464
1,26	13,857395	7	9	7	9	20,452	4,49944
1,26	16,376921	5	8,2	4,2	7	16,99312	4,418211
1,21	15,026296	7,2	11	8	10	23,077	5,07694
1,21	14,343283	7	10,2	8	15	22,39988	4,703975
1,28	15,869141	6,2	9	5	11	18,572	4,82872
1,24	16,909469	6	12,2	4,7	7,8	21,26407	5,528658
1,28	17,089844	6	8	5	8,2	17,593	4,92604
1,3	14,792899	4	6	5	8	15,557	3,88925
-	-	-	-	3,2	4	-	-
1,2	15,277778	5	5,2	-	-	-	-
1,18	15,800057	6	10	4	5	18,572	4,08584
1,32	18,365473	10	16,5	6	7	25,84375	8,27
1,16	14,863258	4	7	15,2	27	25,61908	5,123816
1,22	16,124698	8	11	4	7,2	19,525	4,686
1,42	15,869867	5	6,2	6	10	16,79108	5,373146
1,08	15,432099	8,3	10	5,8	6,8	20,26868	3,648362
1,24	15,608741	6	10	6,2	10	20,63428	4,952227
1,3	16,568047	9	12	7	12	23,077	6,46156
1,26	15,117158	5,7	10	7,8	16	22,05508	5,293219
1,25	19,84	10	16,7	5	13	25,23943	7,824223
1,18	15,800057	10,2	11,8	11,2	25	26,213	5,76686
1,26	19,526329	7,8	12	11	20,7	26,213	8,12603
1,23	18,507502	10,7	12	11	23	26,213	7,33964
1,2	15,277778	8,8	10	12	20	25,468	5,60296
-	-	-	-	5	12	-	-
1,21	15,709309	5	8,2	-	-	-	-
1,32	16,64371	6,7	12,2	5	6	21,53008	6,243723
-	-	-	-	6,7	12	-	-
-	-	-	-	-	-	-	-
1,21	14,343283	5,5	9	-	-	-	-
1,29	15,624061	6,2	8	6	7,2	18,572	4,82872
1,17	14,610271	5,2	7,8	5	7,8	17,39408	3,478816
-	-	-	-	6	8	-	-
-	-	-	-	-	-	-	-

FFM	SC attendance	Weight status	Educational level
18,23938	2	1	2
17,16098	1	1	1
18,76303	1	1	1
18,69302	1	1	1
19,94587	1	1	1
19,57878	1	1	2
22,23	1	1	1
17,91416	1	1	1
17,16699	2	1	1
20,26568	1	1	1
19,13805	2	1	1
14,84259	1	1	1
26,27414	1	2	1
18,04808	1	1	2
21,55563	1	1	1
17,44049	1	1	1
18,12978	2	1	1
19,20198	1	1	2
17,94731	1	1	1
18,64446	1	2	2
19,30258	1	1	-
18,12978	1	1	1
17,60182	1	1	1
17,14056	1	1	1
20,853	1	1	1
18,84028	1	1	1
20,97252	1	1	-
21,37437	1	1	1
18,36198	1	1	1
16,2856	1	1	1
22,46958	1	2	1
16,44182	1	1	1
22,50404	1	1	1
19,54272	1	1	1
25,80806	1	2	1
21,55563	2	1	1
16,4814	1	1	2
21,77616	1	1	1
20,66036	1	2	-
17,30234	1	1	1
18,81	1	1	1
23,08932	1	2	2
19,4942	1	1	1
23,1695	1	2	2
18,83278	1	1	1
19,72864	1	1	1
19,54272	1	1	1
19,93283	1	1	1
16,095	2	1	1

19,86351	1	1	1
24,70539	1	2	2
16,43051	2	1	2
17,51652	1	1	1
-	1	-	1
17,41031	2	1	1
23,70155	1	2	1
21,50531	2	1	-
16,78485	2	1	1
24,1425	1	1	1
14,6265	1	1	1
21,04513	1	1	1
21,17128	1	1	1
19,77768	2	1	1
-	1	-	2
16,38039	1	1	2
19,32632	1	1	1
23,49879	2	1	1
20,357	1	1	-
16,36843	1	1	1
26,03917	1	2	1
-	2	-	-
20,68248	1	1	1
21,19551	1	2	1
19,38196	1	1	1
19,09158	1	1	1
-	1	-	1
20,26568	1	2	1
-	2	-	1
-	1	-	2
-	1	-	2
19,77768	1	1	1
22,14639	1	1	1
24,45205	1	2	1
17,50056	1	1	1
22,50404	1	2	1
22,94143	1	1	1
18,1733	1	1	2
21,75367	1	1	1
21,37637	2	1	1
18,68412	1	1	1
20,57149	2	1	-
-	1	-	1
15,94561	2	1	2
24,6582	1	2	1
18,81	1	1	1
15,19974	2	1	1
16,47868	1	1	1
17,30547	1	1	1
19,30258	1	1	-

18,23938	1	1	1
21,68712	2	1	-
17,3166	1	1	1
24,66903	1	2	1
17,1	2	1	1
27,69228	1	2	1
17,83339	2	1	1
17,503	2	1	1
21,26279	1	1	1
21,37437	1	1	1
22,91952	1	2	1
16,22449	2	1	2
23,41251	2	1	2
23,89892	2	2	2
18,35064	1	1	2
19,54272	2	1	-
27,5025	2	2	1
24,25592	1	1	1
20,5034	1	1	2
22,3036	1	1	1
21,26279	2	2	1
19,33904	1	1	1
-	2	-	1
17,0573	1	1	2
-	1	-	1
20,70848	1	2	1
17,50056	1	1	2
-	2	-	-
22,23702	1	2	1
17,955	1	1	1
20,01888	1	1	2
22,31946	2	2	1
15,63474	2	1	2
17,14056	2	1	1
19,025	1	1	-
20,70147	1	1	1
18,95361	1	-	1
18,46152	2	1	1
20,22072	1	1	2
23,46188	1	1	1
-	2	-	2
18,46613	1	1	1
19,09152	1	1	1
21,0218	1	1	1
18,53164	1	1	1
18,65367	2	1	-
18,85719	1	1	1
18,69302	1	1	1
22,51158	1	1	1
-	1	-	1

-	2	-	-
20,16471	2	1	-
18,15035	2	1	2
18,35064	2	1	2
21,98556	1	1	1
-	1	-	-
-	1	1	1
18,38176	2	1	-
-	2	-	-
-	1	1	1
20,67644	1	1	1
22,58579	1	2	1
22,32477	2	1	-
-	2	-	1
-	1	1	1
20,72656	1	1	2
26,35495	2	2	1
-	1	-	2
-	2	1	-
20,11875	1	1	1
15,8097	1	1	1
-	2	-	1
-	2	1	-
17,14056	1	1	1
24,60337	2	2	-
20,44822	2	1	2
15,38017	2	1	-
-	1	-	-
-	2	1	2
22,02217	2	2	2
23,41721	2	1	2
23,01606	1	2	1
17,09988	1	1	-
14,51943	1	1	1
16,6824	1	1	1
19,13552	1	1	2
22,03077	2	2	2
15,49944	2	1	1
19,49648	2	1	2
21,72825	1	1	1
18,82575	1	1	1
22,18018	1	2	2
15,65172	1	1	1
22,18739	1	1	1
21,75346	2	2	1
16,44182	2	1	1
19,49648	1	1	1
16,52118	1	1	1
17,30547	1	1	1
17,80862	2	1	1

19,42758	2	1	1
26,15382	1	2	1
16,88758	2	1	2
19,09152	1	1	2
20,88295	1	1	1
-	1	-	2
-	2	1	2
16,70508	1	1	1
22,93733	1	1	1
27,20281	1	1	2
22,88131	1	1	1
-	2	1	1
23,95572	2	1	2
19,57878	2	1	1
22,79992	1	1	2
19,12706	1	1	1
18,87528	1	1	1
20,44822	1	1	1
17,09988	1	1	2
21,68712	1	1	1
18,83278	1	1	1
22,533	1	2	2
15,21706	2	1	-
15,88706	1	1	1
19,887	1	1	2
19,76662	2	1	2
19,72836	1	1	1
23,06892	1	1	1
25,026	2	1	1
15,84828	2	1	1
19,49648	1	1	1
18,19168	1	1	1
-	2	-	-
-	1	1	1
21,35351	2	1	1
18,81779	1	-	1
19,04826	1	1	1
26,73998	1	2	1
17,09988	2	1	1
18,50925	2	1	1
17,54089	2	1	1
19,09152	2	1	1
19,04777	2	1	1
20,10951	1	1	1
18,50925	1	1	2
21,77616	1	2	1
18,72844	1	1	1
23,0269	1	2	2
19,04777	2	1	2
-	1	-	2

-	1	1	1
21,93354	1	1	1
17,50056	2	1	1
21,58179	1	1	1
16,92306	1	1	1
16,29603	2	1	1
21,17128	1	1	2
20,47134	1	1	1
23,07396	1	1	1
21,11075	1	1	-
-	2	-	-
-	2	1	1
17,91416	1	1	2
23,73	2	2	-
14,87618	1	1	1
19,314	1	1	1
26,62685	1	1	1
14,35164	1	1	2
19,04777	2	1	1
21,53844	2	1	-
18,70678	2	1	-
23,17578	2	2	-
16,23314	1	1	-
22,87397	2	2	-
20,66036	2	2	-
16,39704	2	1	2
-	2	-	-
-	1	1	1
22,75628	2	1	-
-	-	-	-
-	-	-	2
-	1	1	1
21,17128	2	1	2
16,52118	-	1	-
-	-	-	-
-	-	-	-

S1Appendix file legend

Dichotomized variables in dataset:

Gender: 1 – boy 2 – girl

MVPA norm: 1 – yes 2 – no

SC attendance: 1 – yes 2 – no

Weight status: 1 – normal weight 2 – overweight

Educational level: 1 – university degree 2 – no university degree
